# *Leptospira* spp. strain identification by MALDI TOF MS is an equivalent tool to 16S rRNA gene sequencing and multi locus sequence typing (MLST)

**DOI:** 10.1186/1471-2180-12-185

**Published:** 2012-08-27

**Authors:** Anna Rettinger, Inke Krupka, Karola Grünwald, Viktor Dyachenko, Volker Fingerle, Regina Konrad, Heribert Raschel, Ulrich Busch, Andreas Sing, Reinhard K Straubinger, Ingrid Huber

**Affiliations:** 1 Bacteriology and Mycology, Institute for Infectious Diseases and Zoonoses, Department of Veterinary Sciences, Faculty of Veterinary Medicine, LMU Munich, Veterinaerstr.13, 80539, Munich, Germany; 2 Bavarian Health and Food Safety Authority, Veterinaerstr. 2, D-85764, Oberschleißheim, Germany

**Keywords:** MALDI-TOF MS, *Leptospira interrogans*, *Leptospira kirschneri*, *Leptospira borgpetersenii*, multi locus sequence typing, LipL32, LipL41, rrs2, 16S rRNA, ClinProTools

## Abstract

**Background:**

In this study mass spectrometry was used for evaluating extracted leptospiral protein samples and results were compared with molecular typing methods. For this, an extraction protocol for *Leptospira* spp. was independently established in two separate laboratories. Reference spectra were created with 28 leptospiral strains, including pathogenic, non-pathogenic and intermediate strains. This set of spectra was then evaluated on the basis of measurements with well-defined, cultured leptospiral strains and with 16 field isolates of veterinary or human origin. To verify discriminating peaks for the applied pathogenic strains, statistical analysis of the protein spectra was performed using the software tool ClinProTools. In addition, a dendrogram of the reference spectra was compared with phylogenetic trees of the 16S rRNA gene sequences and multi locus sequence typing (MLST) analysis.

**Results:**

Defined and reproducible protein spectra using MALDI-TOF MS were obtained for all leptospiral strains. Evaluation of the newly-built reference spectra database allowed reproducible identification at the species level for the defined leptospiral strains and the field isolates. Statistical analysis of three pathogenic genomospecies revealed peak differences at the species level and for certain serovars analyzed in this study. Specific peak patterns were reproducibly detected for the serovars Tarassovi, Saxkoebing, Pomona, Copenhageni, Australis, Icterohaemorrhagiae and Grippotyphosa. Analysis of the dendrograms of the MLST data, the 16S rRNA sequencing, and the MALDI-TOF MS reference spectra showed comparable clustering.

**Conclusions:**

MALDI-TOF MS analysis is a fast and reliable method for species identification, although Leptospira organisms need to be produced in a time-consuming culture process. All leptospiral strains were identified, at least at the species level, using our described extraction protocol. Statistical analysis of the three genomospecies *L. borgpetersenii*, *L. interrogans* and *L. kirschneri* revealed distinctive, reproducible differentiating peaks for seven leptospiral strains which represent seven serovars. Results obtained by MALDI-TOF MS were confirmed by MLST and 16S rRNA gene sequencing.

## Background

Leptospirosis is a common mammalian zoonosis occurring worldwide. The causative agents are different serovars of pathogenic *Leptospira* strains, bacteria that belong to the order Spirochaetales. They can affect humans as well as a wide range of different mammals [1] while the clinical manifestations differ considerably [2,3]. In dogs [4-6] and humans [7,8] clinical signs vary from self-limiting flu-like symptoms to a severe illness with manifestation in specific organs, including the kidneys with acute renal failure [9], which can lead to death. In pigs [10,11] and cattle [12] still birth, abortion, and foetal birth deformities may occur. In horses *Leptospira* spp. play a role in the clinical manifestation of the Equine Recurrent Uveitis (ERU) [13].

The systematic classification of *Leptospira* spp. is complex, since the traditional classification is based on the undefined antigenic diversity between serovars [3]. This system divides the genus *Leptospira* in two groups: *Leptospira interrogans* sensu lato including all pathogenic strains and *Leptospira biflexa* sensu lato representing all non-pathogenic and saprophytic strains. Genetic classification is based on DNA hybridization and a wide range of DNA sequencing methods. Twenty genomospecies are currently described [14,15]. Since immunological and genetic typing methods target different cellular structures, these classification systems do not correspond [15]. Consequently, the characterization of *Leptospira* spp. is still challenging and time-consuming. The most commonly used diagnostic tool for clinical samples is antibody detection by the microscopic agglutination test (MAT). If serum antibodies against *Leptospira* spp. are present in a clinical sample, they will agglutinate with viable, cultured organisms of specific *Leptospira* serovars [16]. This test is highly sensitive and specific provided that the panel of bacteria used represents the specific regional epidemiological status regarding pathogenic strains. Furthermore, it is well-described that different outcomes of MAT results can occur when they are performed in different laboratories and with different MAT panels, underlining the need of internal controls [17,18].

Several molecular methods have been established to detect leptospiral DNA using specific targets to trace the agents in clinical samples such as urine. However, recently developed PCR protocols using different target genes are able to distinguish only between pathogenic and non-pathogenic leptospiral strains [19-22].

Nevertheless, in the past years it has been shown that mass spectrometry is a reliable tool for bacterial identification [23]. Matrix assisted laser desorption ionization-time of flight mass spectrometry (MALDI-TOF MS) is a fast and easily applied method for bacteria classification at the species level [23-25]. Mass spectrometry detects and compares individual protein mass peaks of bacterial cells. Samples can either be spotted as native bacteria cells (direct smear), or an additional extraction step can be performed to purify the proteins of the bacteria. Most studies so far were performed with bacterial colonies grown on various solid agar-based media or MALDI-TOF MS was used to identify microorganisms directly in clinical samples such as blood or urine [26]. Only a few studies describe the mass spectrometry analysis for bacteria grown in liquid media [27,28]. This can be critical regarding the methodical MALDI-TOF MS sample preparation, and can limit the application for bacteria such as *Borrelia* or *Leptospira*, which are commonly grown in nutrient enriched semisolid or liquid media [29].

Recently, it was shown that directly spotted *Leptospira* samples can be identified at the species level using MALDI-TOF MS [27]. For some bacterial groups, it has been reported that extracted samples allow better identification than directly smeared samples [30-32]. This is due to the better quality achieved with extracted samples. In this study we, therefore, evaluated the use of MALDI-TOF MS for extracted *Leptospira* strains and compared our results with molecular typing methods. The extraction protocol established in this study for *Leptospira* spp. grown in liquid media was used to create a reference spectra database of 28 well-defined *Leptospira* strains. Based on multiple measurements, the database was evaluated with characterized leptospiral strains and with 16 field isolates. Statistical analysis with two independently compiled datasets of *L. interrogansL. borgpetersenii* and *L. kirschneri* was performed to visualise peak pattern differences of the protein spectra at species level and for certain serovars used in this approach. To confirm the identity for all tested strains, 16S rRNA sequencing and multi locus sequence typing (MLST) analysis was performed and compared to a created dendrogram containing all established reference spectra. In conclusion, MALDI-TOF MS is a rapid and easily applicable method for the characterisation of *Leptospira* spp. at the species level, and differentiating peaks were identified for a number of the examined strains indicating serovar affiliation. The method can be used as a comparable tool to well-established molecular genetic typing methods like MLST.

## Methods

### Bacterial strains and culture conditions

A panel of 28 leptospiral reference strains was used in this study, including pathogenic, non-pathogenic and intermediate strains (Table 1). The pathogenic strains represent the main causative serovars for Leptospirosis in humans and animals. The most common strains used in MAT panels belong to the three genomospecies *L. interrogans*, *L. borgpetersenii* and *L. kirschneri* (Table 1). All strains were cultured in Ellinghausen-McCullough-Johnson-Harris medium (Leptospira Medium Base EMJH BD, Difco^TM^ and Leptospira Enrichment EMJH Difco^TM^, NJ, USA) at 28°C. Cultures were controlled for growth and motility by darkfield microscopy and were periodically subcultured into fresh media. Bacteria used for MALDI-TOF MS measurements and protein reference spectra generation were cultured for seven days.

**Table 1:** ***Leptospira*reference strains used for MALDI-TOF MS measurements and sequence analysis**

**genomospecies**	**serogroup**	**serovar**	**strain**	**pathogenicity**
*L. interrogans*	Australis	Australis	Ballico ^a, b^	pathogenic
*L. interrogans*	Australis	Bratislava	Jez Bratislava ^a, b^	pathogenic
*L. interrogans*	Autumnalis	Autumnalis	Akiyami A ^a, b^	pathogenic
*L. interrogans*	Bataviae	Bataviae	Swart ^a, b^	pathogenic
*L. interrogans*	Canicola	Canicola	Hond Utrecht IV ^a, b^	pathogenic
*L. interrogans*	Hebdomadis	Hebdomadis	Hebdomadis ^a, b^	pathogenic
*L. interrogans*	Icterohaemorrhagiae	Copenhageni	M 20 ^a, b^	pathogenic
*L. interrogans*	Icterohaemorrhagiae	Icterohaemorrhagiae	Ictero I ^b^	pathogenic
*L. interrogans*	Pomona	Pomona	Pomona ^a,b^	pathogenic
*L. interrogans*	Pyrogenes	Pyrogenes	Salinem ^a,b^	pathogenic
*L. interrogans*	Sejroe	Hardjo	Hardjoprajitno ^a,b^	pathogenic
*L. kirschneri*	Grippotyphosa	Grippotyphosa	Moskva V ^a.b^	pathogenic
*L. borgpetersenii*	Sejroe	Saxkoebing	Mus 24 ^a, b^	pathogenic
*L. borgpetersenii*	Ballum	Ballum	Mus 127 ^a, b^	pathogenic
*L. borgpetersenii*	Sejroe	Sejroe	M 84 ^a, b^	pathogenic
*L. borgpetersenii*	Tarassovi	Tarassovi	Perepelitsin ^a, b^	pathogenic
*L. borgpetersenii*	Javanica	Javanica	Veldrat Bataviae 46 ^b^	pathogenic
*L. alexanderi*	not defined	Manhao 3	L60^c^	pathogenic
*L. weilii*	not defined	Celledoni	Celledoni ^c^	pathogenic
*L. santarosai*	not defined	Shermani	LT 821 ^c^	pathogenic
*L. noguchii*	not defined	Panama	CZ 214 ^c^	pathogenic
*L. broomii*	not defined	Not defined	5399 ^c^	intermediate
*L. fainei*	not defined	Hurstbridge	BUT 6 ^c^	intermediate
*L. inadai*	not defined	Lyme	10 ^c^	intermediate
*L. biflexa*	Semaranga	Patoc	PatocI ^c^	non-pathogenic
*L. meyeri*	not defined	Semaranga	Veldrat S173 ^c^	non-pathogenic
*Turneriella parva*	not defined	Parva	H ^c^	non-pathogenic
*Leptonema illini*	not defined	Illini	3055 ^c^	non-pathogenic

^a^ Acquired by purchase at the Federal Institute for Risk Assessment (BfR) Head of Unit. Diagnostics, Genetics and Pathogen Characterisation, Department Biological Safety Berlin, Germany.

^b^ Acquired by purchase at the WHO/FAO Collaborating Centre for Reference and Research on Leptospirosis, Biomedical Research, Royal Tropical Institute (KIT) Amsterdam, The Netherlands.

^c^ Acquired by purchase at the DSMZ - German Collection of Microorganisms and Cell Cultures, Braunschweig, Germany.

### Sample preparation for MALDI-TOF MS measurements

After seven days of growth and diluting the cultures with Ellinghausen-McCullough-Johnson-Harris medium (EMJH media) 1:10 or 1:100 depending on the number of cells, bacteria were counted using a Petroff Hausser counting chamber (HS Hausser Scientific, Horsham, PA) by darkfield microscopy. For further preparation steps, the concentration of bacteria needed to be at least 1 x 10^6^ organisms per ml.

For ethanol/formic acid extraction, 1 ml of culture was centrifuged at 14.000 rpm at room temperature for 10 minutes. The supernatant was removed and the pellet was suspended in 300 μl distilled water. The suspension was then vortexed until the pellet was completely dissolved. Nine hundred microliters of ethanol (Roth, Rotipan® ≥ 99, 8% p.a., Karlsruhe, Germany) was added to inactivate the microorganisms, followed by vortexing of the suspension. After centrifugation for 10 min at 14.000 rpm at room temperature, the pellet was visible as a grey layer on the wall of the tube. Samples were air-dried, or dried in a concentrator for 10 min at 30°C (Concentrator plus, Eppendorf AG, Hamburg, Germany) to ascertain that the ethanol could evaporate completely. The material was then dissolved in 30 μl of 70% formic acid (Merck, 98–100%, Darmstadt, Germany) followed by addition of 30 μl acetonitrile (Fluka Analytical Sigma-Aldrich, Munich, Germany). It has to be pointed out that equal volumes of 70% formic acid and acetonitrile were applied. Again, centrifugation was performed at 14.000 rpm for 2 min at room temperature. One microliter of the clear supernatant was spotted on a MSP 96 target polished steel plate (Bruker Daltonik GmbH, Bremen, Germany) and allowed to dry. Following this, the dried spot was overlaid with 1 μl of matrix solution, a saturated solution of α-Cyano-4-hydroxycinnamic acid (HCCA, 99% Bruker Daltonik GmbH, Bremen respectively Sigma-Aldrich, Munich, Germany) composed of 50% acetonitrile (Fluka Analytical Sigma-Aldrich) and 2.5% triflouracetic acid (TFA Reagent Plus® 99% 100 ml, Sigma-Aldrich). Finally, samples were allowed to dry at room temperature. An optional washing step was included into the extraction protocol, to investigate if this influenced the quality of the protein spectra measurements. This step was carried out once after the first centrifugation of the cultured material with 200 μl phosphate buffered saline (PBS) and centrifuged again for 10 min at 14.000 rpm at room temperature.

### MALDI-TOF MS instrumental settings

Measurements were performed with two different MALDI-TOF MS instruments in two laboratories. In both cases, the Microflex LT System, MALDI Biotyper™ (Bruker Daltonik GmbH, Bremen, Germany), equipped with a 60-Hz nitrogen laser was employed, using the Software for FLEX Series 1.3. Spectra were recorded in a linear positive ion detection mode in a mass range from 2,000 to 20,137 Da. Spectrometer settings were set to: Ion Source 1 (IS1) 20 kV; Ion source 2 (IS2) 16.69 kV; Lens voltage: 7 kV; Pulsed Ion Extraction: 150 ns. Each spectrum was created with the software Flex Control (Version 3.3) either in an automatic mode with variable laser power or manually with a laser power set between 29–33%. For each spectrum a total of 240 shots were summed up. Before each measurement, the instrument was calibrated using the bacterial test standard (BTS), an *Escherichia coli DH5alpha* extract, spiked with two additional proteins (RNase A and myoglobine) provided by Bruker Daltonik GmbH (Bremen, Germany). Preparation of the BTS and calibration were performed following the manufacturer’s instructions. Calibration was successful when proteins of the mass spectra were in a range of ± 300 ppm (parts per million).

### Protein reference spectra creation and MALDI-TOF MS measurements

For the 28 leptospiral reference strains (Table 1) reference spectra, in the following called MSPs (main spectral projections), were created independently in two different laboratories. Main spectra represent individual protein spectra for one bacterial strain. To achieve representative results, at least 20 individual spectra were used to create a single MSP as proposed by Bruker Daltonik GmbH (Bremen, Germany). Each sample was spotted on eight positions of the target and 24 to 30 raw spectra of the leptospiral strain and one spectrum of the bacterial test standard were measured automatically with the Flex control software. Spectra were analyzed with the Flex Analysis software (Version 3.3). The BTS was used for internal calibration. In a second step the uniformity of the created spectra sets was visually checked in a mass range of 3,000 Da to 10,000 Da. Spectra with peaks outside the allowed average were removed. Modified spectra were loaded into the MALDI BioTyper™ 3.0 Version (Bruker Daltonik GmbH, Bremen, Germany). Software settings for MSP creation were set to: maximal mass error of each single spectrum: 2,000; desired mass error for the MSP: 200; desired peak frequency minimum (%): 25; maximal desired peak number of the MSP: 70. Reference spectra were created automatically by the software and all created spectra were added to the main spectra library as unassigned MSPs.

The created reference spectra were evaluated based on measurements with the defined strains (see Table 1) and, additionally, with 16 field isolates (Table 2). Each strain was prepared using the ethanol/formic acid extraction and spotted on the target. Each spot was measured twice in both automatic and manual modes on different target spots.

**Table 2:** **16*Leptospira*field isolates identified by MALDI-TOF MS measurements and 16S rRNA sequencing**

**genomospecies**	**serogroup**	**serovar**	**strain number**	**origin**
*L. borgpetersenii*	Sejroe	Saxkoebing	LGL 489	corpus vitreum, horse
*L. interrogans*	Australis	Australis	LGL 537	corpus vitreum, horse
*L. interrogans*	Australis	Bratislava	LGL 538	corpus vitreum, horse
*L. interrogans*	Icterohaemorrhagiae	Icterohaemorrhagiae	LGL 113	human urine
*L. interrogans*	Icterohaemorrhagiae	Icterohaemorrhagiae	LGL 535	human urine
*L. interrogans*	Icterohaemorrhagiae	Icterohaemorrhagiae	LGL 540	corpus vitreum, horse
*L. interrogans*	Icterohaemorrhagiae	Icterohaemorrhagiae	LGL 471	human blood
*L. interrogans*	Canicola	Canicola	LGL 87	human urine
*L. kirschneri*	Grippotyphosa	Grippotyphosa	LGL 517	corpus vitreum, horse
*L. kirschneri*	Grippotyphosa	Grippotyphosa	LGL 518	corpus vitreum, horse
*L. kirschneri*	Grippotyphosa	Grippotyphosa	LGL 533	corpus vitreum, horse
*L. kirschneri*	Grippotyphosa	Grippotyphosa	LGL 539	corpus vitreum, horse
*L. kirschneri*	Grippotyphosa	Grippotyphosa	LGL 541	corpus vitreum, horse
*L. kirschneri*	Grippotyphosa	Grippotyphosa	LGL 112	human urine
*L. kirschneri*	Pomona	Pomona	LGL 511	corpus vitreum, horse
*L. kirschneri*	Pomona	Pomona	LGL 532	corpus vitreum, horse

Spectra loaded into MALDI BioTyper™ 3.0 Version were measured at the default settings. Unknown spectra were compared with the created reference library by using a score value, the common decadal logarithm for matching results.

Results were analyzed following the score value system according to Bruker Daltonik GmbH (Bremen, Germany). Values from 3.00 to 2.30 indicate reliable species identification; values from 2.29 to 2.00 indicate reliable genus identification and probable species identification. Lower values stand for probable genus identification or no reliable match with the MSP database (http://www.bdal.de).

### Statistical analysis using the ClinProTools software

MALDI-TOF MS spectra were exported into ClinProTools software version 2.2 (Bruker Daltonik GmbH, Bremen, Germany) to carry out statistical analysis. The software was used for visual comparison of the loaded spectra, as well as for identifying specific peaks of interest. First, 20 spectra for each of the investigated strains were loaded into the program and were automatically recalibrated. To compare individual strains, the same numbers of protein spectra were required to be analyzed using ClinProTools. Classification models were automatically generated. For this, the specific algorithms of the software, including QuickClassifier (QC)/Different Average, Supervised Neural Network (SNN) and the Genetic Algorithm were used. These algorithms proposed a list of discriminating peaks for the analyzed spectra according to the selected algorithm. Suggested peaks were visually evaluated and compared with the original spectra. This procedure was done for all algorithms and a manual report was created with the most relevant and reproducible mass peaks. Furthermore, statistical testing of the datasets was performed on the basis of principle component analysis (PCA) and results were displayed in a three-dimensional score plot, which was generated automatically by the software.

### Genotyping

Strain confirmation was performed by sequencing all strains on the basis of a multi locus sequence typing as described by Ahmed et al. [33]. This MLST is based on six target genes: *secY* (pre-protein translocase SecY protein), *rrs2* (16s rRNA), *adk* (adenylate kinase), *icdA* (isocitrate dehydrogenase), *LipL32* and *LipL41* (outer membrane lipoproteins). All leptospiral strains were aligned to reference sequences for the six genes in the NCBI GenBank, if adequate sequences were available. Accession numbers for *L. interrogans* serovar Copenhageni strain Fiocruz L1-130 are AE016823.1 and for *L. borgpetersenii* serovar Hardjo-bovis strain L550: CP000348.1. Accession numbers for the *Treponema* outgroup are AE017226.1, CP001843.1 and CP000805.1. For DNA extraction, each strain was cultured for seven days. Six millilitres of the cultured organisms were centrifuged at 14.000 rpm, 4°C for 10 min, the pellet was then washed once with PBS and either stored at −30°C or used directly for DNA extraction. Extraction was performed using the QIAamp® DNA Mini Kit (Qiagen, Hilden, Germany) following the manufacturer’s instructions. PCR for each target gene was performed using 25 mM MgCl_2_ (included in the 10x standard reaction buffer, NEB, Frankfurt am Main, Germany)_,_ 0.2 mM dNTP`s (NEB), 1 U *Taq* DNA Polymerase (NEB) and 1 μl template DNA. Amplification parameters were set according to Ahmed et al. [33], using the Master Cycler® pro system (Eppendorf AG, Hamburg, Germany). PCR products were visualized in 1.6% agarose gels. Products were then purified using the peqGOLD Gel Extraction Kit (Peqlab, Erlangen, Germany) following the manufacturer’s instruction. Five nanograms per μl of the purified product were sequenced by Eurofins MWG Operon (Ebersberg, Germany). All strains were sequenced twice. Sequence analysis was performed by using the MEGA4 Software and Neighbor Joining trees were constructed for each gene and for each leptospiral strain according to Ahmed et al. [33].

### 16S rRNA gene sequencing

16S rRNA gene sequencing was performed with the bacterial universal primers 27f (agagtttgatcmtggctcag) and 1392r (acgggcggtgtgtgtrc) (see GATC Biotech AG, Konstanz, Germany; http://www.gatc-biotech.com, free universal primers). PCR was performed using HotStarTaq® Master Mix (Qiagen, Hilden, Germany) with the following profile: 15 min at 95°C for initial denaturation, 35 cycles of 30 sec at 95°C, 30 sec at 56°C and 1.5 min at 72°C, followed by a final extension step of 72°C for 5 min. PCR products were purified using the QIAquick PCR purification kit (Qiagen, Hilden, Germany) and sequence analyses were performed using the Cycle Sequencing Kit (Applied Biosystems, Carlsbad, California, USA) following the manufacturer’s instructions. Sequencing was carried out on Applied Biosystems 3130 Genetic Analyzer (Applied Biosystems, Carlsbad, California, USA) and the sequences were analyzed using the 16S rRNA gene database of SmartGene (Lausanne, Switzerland). A Maximum Likelihood phylogenetic tree of all 28 leptospiral 16S rRNA gene sequences was computed with PHYLIP dnaml (SmartGene). The EMBL accession numbers for the 28 leptospiral 16S rRNA gene sequences were as follows: JQ988836, JQ988837, JQ988838, JQ988839, JQ988840, JQ988841, JQ988842, JQ988843, JQ988844, JQ988845, JQ988846, JQ988847, JQ988848, JQ988849, JQ988850, JQ988851, JQ988852, JQ988853, JQ988854, JQ988855, JQ988856, JQ988857, JQ988858, JQ988859, JQ988860, JQ988861, JQ988862, JQ988863. The accession number for *Treponema pallidum* was AE000520.

## Results

### Sample extraction procedure and MALDI-TOF MS measurements

This study focused mainly on well-defined pathogenic leptospiral strains used for serodiagnostic purposes which belong to three genomospecies: *L. interrogans*, *L. borgpetersenii* and *L. kirschneri*. To complete the strain collection, analyses were also performed with intermediate and non-pathogenic strains (see Table 1).

To assess the influence of the optional washing step in the sample preparation procedure for MALDI-TOF MS typing, regarding the quality of the protein spectra, we compared strains that were prepared with and without the optional additional washing step combined with the concentrator process. No differences were found in the created protein spectra when the concentrator was used to evaporate the ethanol. However, the use of the concentrator shortened the vaporizing step to 10 minutes. When the PBS washing step was omitted, peaks representing protein sizes larger than 11,000 Da were removed (data not shown). No differences were seen for reference spectra that were created on two different MALDI-TOF MS instruments (data not shown). To evaluate if the number of passages showed any influence on the quality of the protein spectra, measurements of all reference strains were applied, with cultures that were cultivated up to thirteen passages. The number of passages did not show any influence on the quality of the protein spectra (data not shown).

### Reference spectra database creation for MALDI-TOF MS

**Figure 1:**
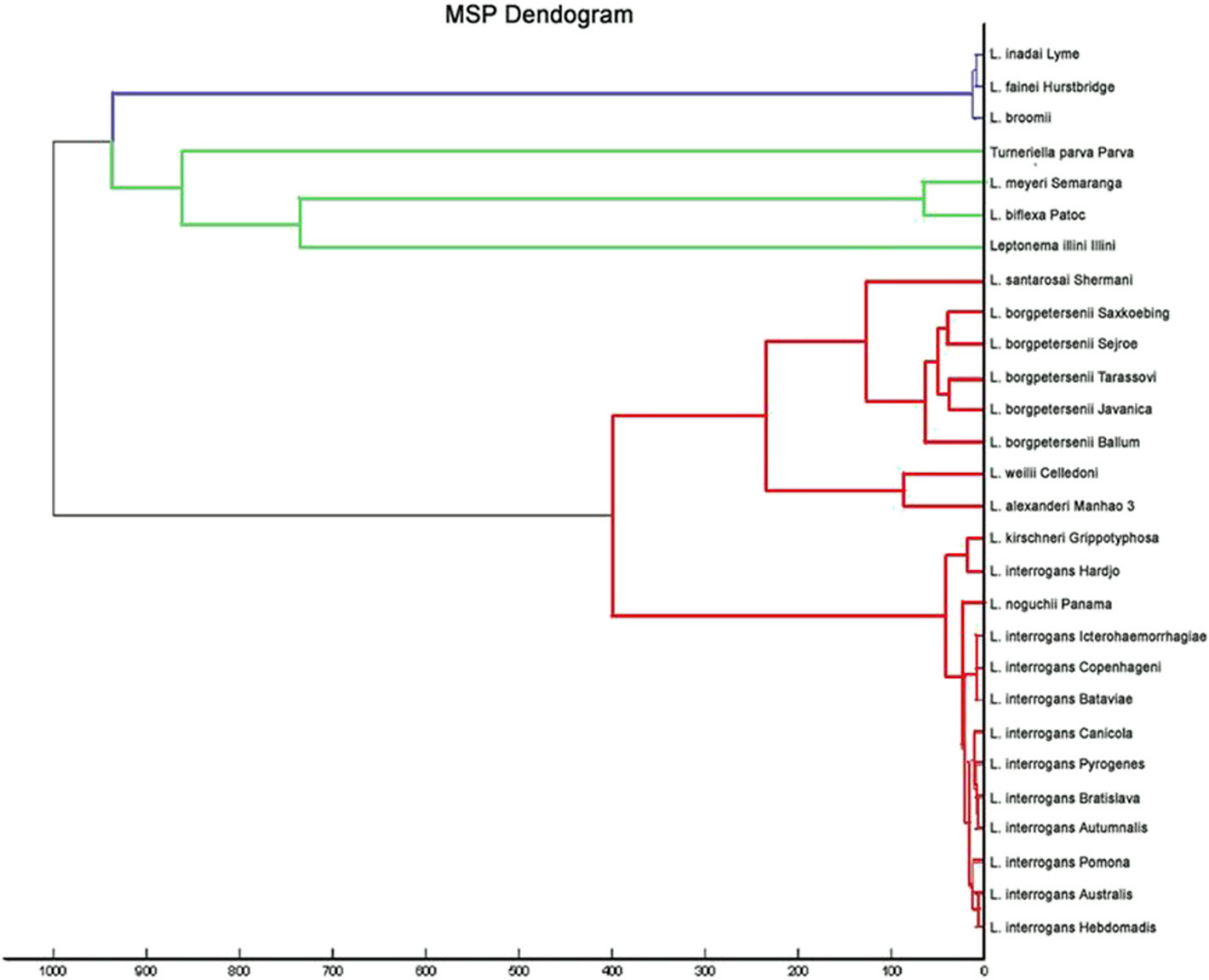
**Dendrogram representing the protein reference spectra of the 28 leptospiral strains.** blue: intermediate leptospiral strains green: non-pathogenic leptospiral strains red: pathogenic leptospiral strains.

Since the commercially available MALDI Biotyper™ database lacks leptospiral protein profiles, reference spectra were created for all 28 leptospiral strains listed in Table 1. The established database was implemented in the reference spectra library as unassigned MSPs. Using the software MALDI Biotyper™ all 28 leptospiral protein reference spectra were visualized in a dendrogram (Figure 1). Each of the 28 strains yielded a species-specific protein profile and was clustered according to its pathogenicity in the MALDI-TOF MS dendrogram. The strains of the pathogenic *Leptospira* species (red color) could clearly be differentiated from the non-pathogenic *Leptospira* species (green color) as well as from the intermediate species (blue color). Within the pathogenic species *L. borgpetersenii* and *L. interrogans* were located in separate clusters. Discrimination was difficult for the species *L. interrogans* and *L. kirschneri* (see Figure 1).

### Protein spectra database evaluation

To test whether the established reference spectra can be used for *Leptospira* species identification, analyses were first performed with the well-defined leptospiral strains from Table 1. Measurements were repeated at least four times for each leptospiral strain to achieve reproducible results. All samples, including the non-pathogenic and intermediate strains, were correctly assigned at the species level. All reference strains of *L. interrogans* and the closely related strain of *L. kirschneri* serovar Grippotyphosa matched with the correct genomospecies at first place.

In addition, 16 leptospiral field isolates (Table 2) were identified with the MALDI-TOF MS (Table 3). Field isolates belonging to one single *L. borgpetersenii* serovar and the seven *L. interrogans* strains matched with the correct genomospecies. Seven field isolates of the genomospecies *L. kirschneri* were also grouped within the correct species. One *L. kirschneri* isolate (LGL strain number 518) matched with the same score value of 2.18 in two different measurements with *L. kirschneri* and *L. interrogans* (marked with ^a^ in Table 3). 16S rRNA sequencing of all field isolates confirmed the MALDI-TOF results with a clear species identification of LGL strain 518 as *L. kirschneri*. Applying MALDI Biotyper ^TM^ identification it was not possible to differentiate the leptospiral strains below the species level.

**Table 3:** Identification results of the 16 leptospiral field isolates by MALDI-TOF MS and 16S rRNA gene sequencing

**field isolate (LGL strain number)**	**MALDI-TOF MS**		**gene sequencing (16S rRNA)**
	**first match**	**score value**	
*L. interrogans* Canicola (87)	*L. interrogans* Hebdomadis	2.62	*L. interrogans*
*L. interrogans* Bratislava (538)	*L. interrogans* Bratislava	2.37	*L. interrogans*
*L. interrogans* Bratislava (540)	*L. interrogans* Autumnalis	2.46	*L. interrogans*
*L. interrogans* Australis (537)	*L. interrogans* Hardjo	2.54	*L. interrogans*
*L. interrogans* Icterohaemorrhagiae (113)	*L. interrogans* Icterohaemorrhagiae	2.67	*L. interrogans*
*L. interrogans* Icterohaemorrhagiae (471)	*L. interrogans* Icterohaemorrhagiae	2.54	*L. interrogans*
*L. interrogans* Icterohaemorrhagiae (535)	*L. interrogans* Icterohaemorrhagiae	5.57	*L. interrogans*
*L. kirschneri* Grippotyphosa ^a^ (518)	*L. kirschneri* Grippotyphosa	2.18	*L. kirschneri*
*L. kirschneri* Grippotyphosa ^a^ (518)	*L. interrogans* Canicola	2.18	*L. kirschneri*
*L. kirschneri* Grippotyphosa (517)	*L. kirschneri* Grippotyphosa	2.38	*L. kirschneri*
*L. kirschneri* Grippotyphosa (533)	*L. kirschneri* Grippotyphosa	2.09	*L. kirschneri*
*L. kirschneri* Grippotyphosa (541)	*L. kirschneri* Grippotyphosa	2.13	*L. kirschneri*
*L. kirschneri* Grippotyphosa (112)	*L. kirschneri* Grippotyphosa	2.54	*L. kirschneri*
*L. kirschneri* Grippotyphosa (539)	*L. kirschneri* Grippotyphosa	2.17	*L. kirschneri*
*L. kirschneri* Pomona (532)	*L. kirschneri* Grippotyphosa	2.28	*L. kirschneri*
*L. kirschneri* Pomona (511)	*L. kirschneri* Grippotyphosa	2.34	*L. kirschneri*
*L. borgpetersenii* Saxkoebing (489)	*L. borgpetersenii* Saxkoebing	2.49	*L. borgpetersenii*

The table pictures the most reliable identification of the field isolates with the established MSP database and lists the achieved score values.

^a^*L. kirschneri* isolate that matched with the reference strain of *L. interrogans* as well as with *L. kirschneri* at first place.

### Detection of differentiating peaks within the pathogenic genomospecies

This analysis was performed to attempt the identification of discriminating peaks for serovars used in this study. For this, both datasets of the two institutions were analyzed by using the statistical software ClinProTools (Bruker Daltonik GmbH, Bremen, Germany). Datasets of the genomospecies *L. interrogans*, *L. kirschneri* and *L. borgpetersenii* were screened for analogies and differences in their protein profile peak patterns in order to identify specific peaks that would allow the discrimination of the analyzed serovars. As *L. interrogans* and *L. kirschneri* showed a very close relationship at the species level, these two genomospecies were analyzed independently from the species *L. borgpetersenii*.

The individual strains were analyzed applying different algorithms of the software. For this, the software selects peak combinations, which are most relevant for the separation of the analyzed dataset. Within the species *L. interrogans* individual protein peak sets were present for the serovar Pomona (3,206 Da, 3,220 Da and 3,234 Da) and the serovar Copenhageni (3,636 and 3,657 Da), resulting in visually unique peak patterns. In addition, individual peak patterns were present for the serovars Australis, and Icterohaemorrhagiae. Beyond that, it was possible to discriminate *L. kirschneri* serovar Grippotyphosa from *L. interrogans* strains with an individual protein peak at 8,097 Da (see Table 4). To ascertain whether strains within the *L. kirschneri* species display different peak patterns, the protein spectra (MSP) of one field isolate of the serovar Pomona (LGL 511, see Table 2) was added to the dataset. Comparison of the two *L. kirschneri* serovars showed a mass deviation from 8,097 Da to 8,081 Da for the *L. kirschneri* Pomona field isolate (data not shown). Discriminating peaks occurred also within the species *L. borgpetersenii* (Table 5). The serovars Saxkoebing and Tarassovi were separated by individual protein peaks at 7,547 Da and 5,765 Da and showed a unique protein pattern each (see Table 5).

**Table 4:** **Differentiating peaks based on the statistical analysis of ClinProTools within the species*L. interrogans*and*L. kirschneri***

**genomospecies**	**peak mass (m/z) representing the protein size in Dalton**									
	**3,206**	**3,220**	**3,234**	**3,636**	**3,657**	**5,526**	**6,191**	**6,327**	**7,358**	**8,097**
*L. interrogans* Hebdomadis	**-**	**-**	**-**	**-**	**-**	**+**	**+**	**-**	**+**	**-**
*L. interrogans* Australis	**-**	**-**	**-**	**-**	**-**	**-**	**+**	**-**	**+**	**-**
*L. interrogans* Autumnalis	**-**	**-**	**-**	**-**	**-**	**+**	**+**	**-**	**+**	**-**
*L. interrogans* Bratislava	**-**	**-**	**-**	**-**	**-**	**+**	**+**	**-**	**+**	**-**
*L. interrogans* Canicola	**-**	**-**	**-**	**-**	**-**	**+**	**+**	**-**	**+**	**-**
*L. interrogans* Copenhageni	**-**	**-**	**-**	**++**	**++**	**+**	**+**	**+**	**-**	**-**
*L. interrogans* Hardjo	**-**	**-**	**-**	**-**	**-**	**+**	**+**	**-**	**+**	**-**
*L. interrogans* Pomona	**++**	**++**	**++**	**-**	**-**	**+**	**+**	**+**	**-**	**-**
*L. interrogans* Pyrogenes	**-**	**-**	**-**	**-**	**-**	**+**	**+**	**-**	**+**	**-**
*L. interrogans* Icterohaemorrhagiae	**-**	**-**	**-**	**-**	**-**	**+**	**+**	**+**	**-**	**-**
*L. interrogans* Bataviae	**-**	**-**	**-**	**-**	**-**	**+**	**+**	**-**	**+**	**-**
*L. kirschneri* Grippotyphosa	**-**	**-**	**-**	**-**	**-**	**+**	**-**	**-**	**+**	**+**

The displayed peaks are based on visual comparison of the algorithms analysis results of the software. All strains were screened twice using the QuickClassifier (QC)/Different average and SNN algorithms.

The used symbols stand for:

no peak found: -

peak present: +

peak set with high intensity: ++

**Table 5:** **Differentiating peaks based on the statistical analysis of ClinProTools within the species*L. borgpetersenii***

**genomospecies**	**peak mass (m/z) representing the protein size in Dalton**					
	**3,759**	**5,765**	**5,779**	**6,388**	**7,519**	**7,547**
*L. borgpetersenii* Ballum	**+**	**-**	**+**	**-**	**+**	**-**
*L. borgpetersenii* Javanica	**+**	**-**	**+**	**-**	**+**	**-**
*L. borgpetersenii* Sejroe	**+**	**-**	**+**	**-**	**+**	**-**
*L. borgpetersenii* Saxkoebing	**-**	**-**	**+**	**+**	**-**	**+**
*L. borgpetersenii* Tarassovi	**+**	**+**	**-**	**+**	**+**	**-**

The displayed peaks are based on visual comparison of the algorithm analysis results of the software. All strains were screened twice using the QuickClassifier (QC)/Different average and SNN algorithms.

The used symbols stand for:

no peak found: -

peak present: +

**Figure 2:**
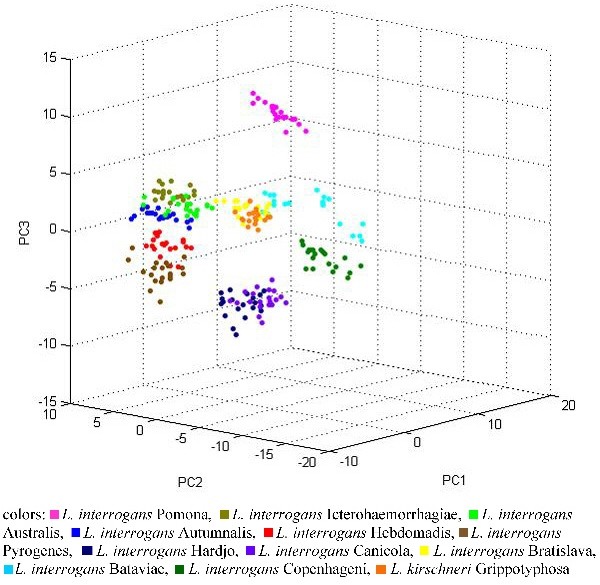
**Principle Component Analysis (PCA) of the analyzed strains of the genomospecies.***L. interrogans* and *L. kirschneri* using the software tool ClinProTools.

**Figure 3:**
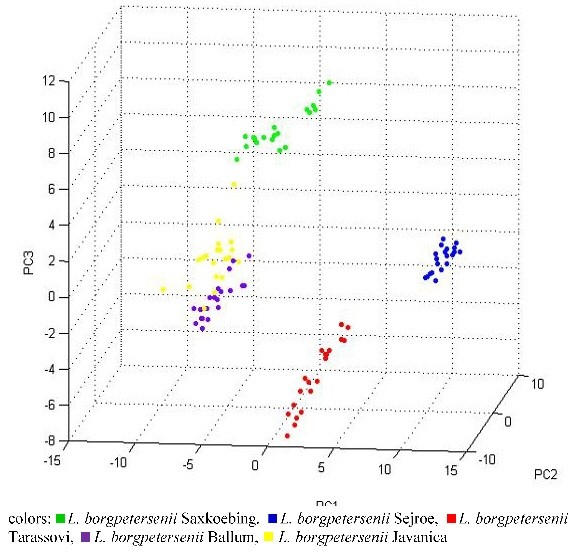
**Principle Component Analysis (PCA) of the analyzed strains of the genomospecies.***L. borgpetersenii* using the software tool ClinProTools.

The additional statistical tool Principal component analysis (PCA) included in ClinProTools was applied to the analyzed datasets to visualize the homogeneity and heterogeneity of the protein spectra. PCA reduces the variables of a complex dataset on the basis of different statistical tests. The reduced datasets, the so-called PCs (principle components) can be displayed in a score plot illustration. Twenty individual protein spectra of the *L. interrogans* strains and the *L. kirschneri* strain are displayed in three-dimensional PCA in Figure 2. Each dot stands for a displayed protein spectrum. The colors indicate the calculated cluster membership in which each dot represents one measured protein spectrum profile for each sample. A clear separation of the serovars Pomona and Copenhageni is apparent. Conversely, *L. kirschneri* serovar Grippotyphosa did not cluster separately in PCA analysis, even if specific peaks could be detected for *L. kirschneri* in the peak statistics (see Table 4). For the genomospecies *L. borgpetersenii* the separation of the serovars Saxkoebing, Sejroe and Tarassovi was apparent when PCA was performed (Figure 3).

### Strain confirmation and molecular sequencing

**Figure 4:**
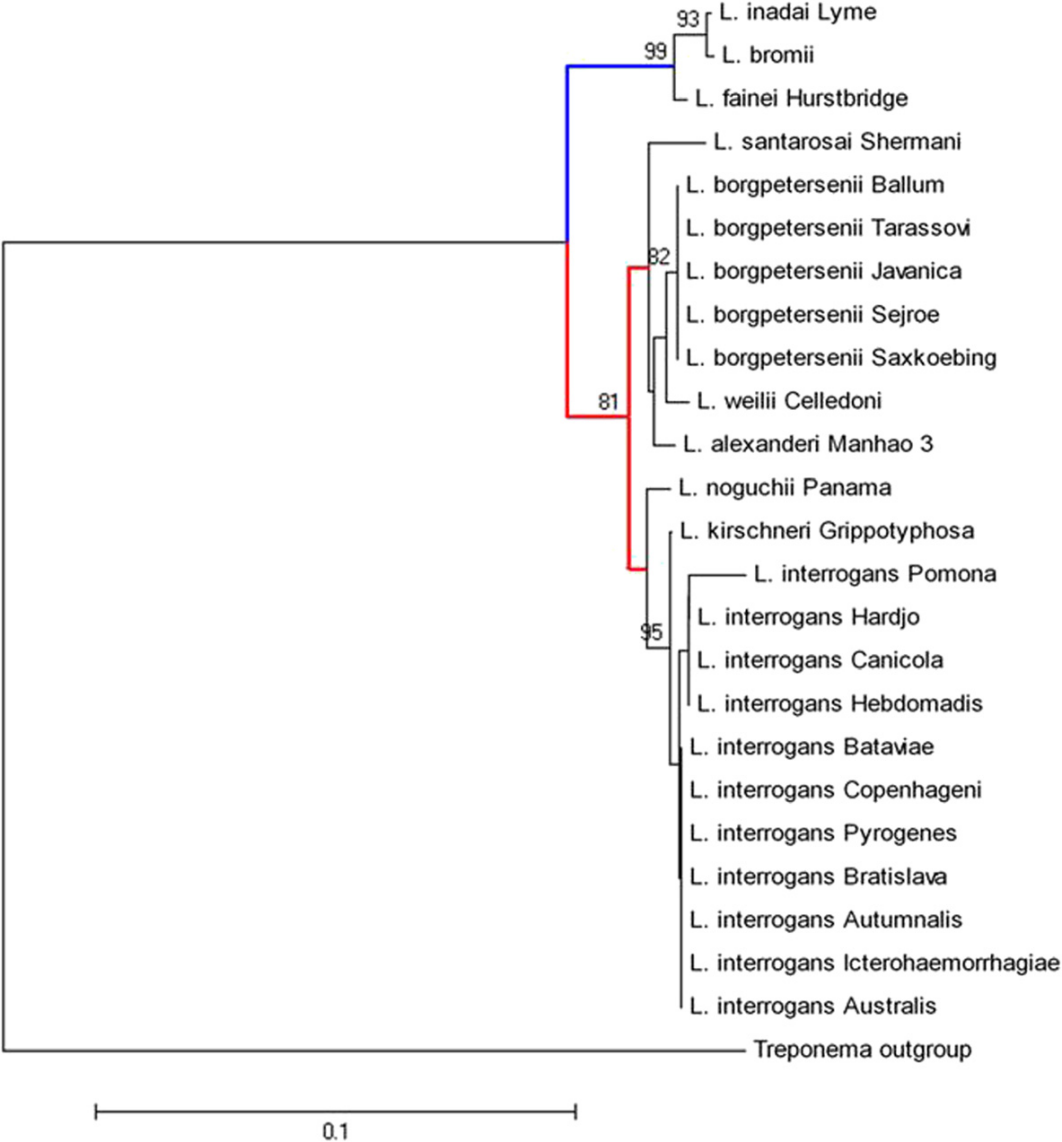
**Neighbor Joining tree based on multi locus sequence typing analysis.** The bar indicates 0.1 estimated substitution per sequence position. blue: intermediate leptospiral strains, red: pathogenic leptospiral strains.

**Figure 5:**
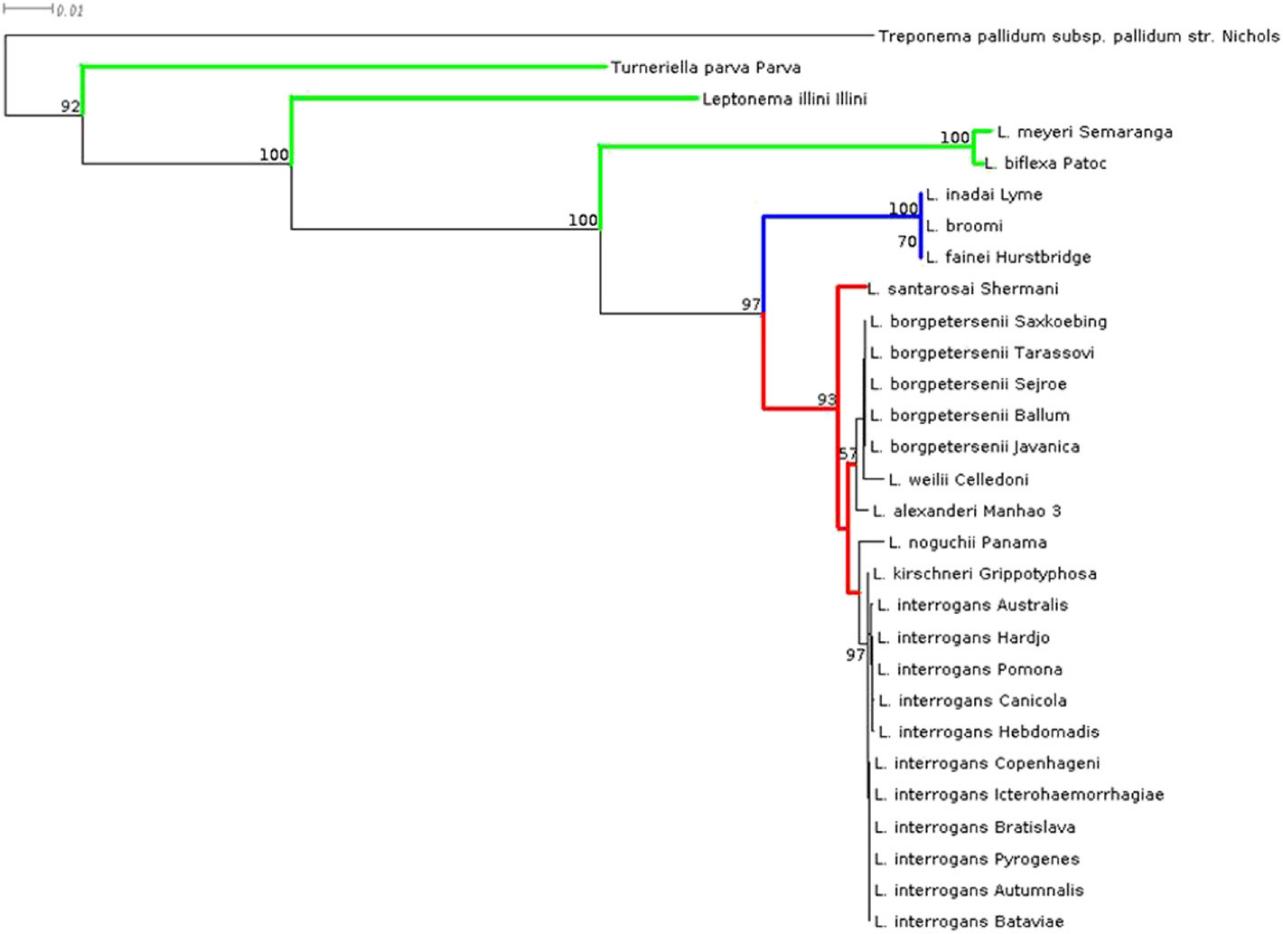
**Maximum Likelihood phylogenetic tree based on the 16S rRNA sequencing.** The bar indicates 0.01 estimated substitution per sequence position. blue: intermediate leptospiral strains, green: non-pathogenic leptospiral strains, red: pathogenic leptospiral strains.

Sequence analysis of the 28 leptospiral reference strains was performed on the basis of MLST analysis (Figure 4) and 16S rRNA gene sequencing (Figure 5). Confirmation of the field isolates relied on 16S rRNA gene sequencing. Species identity of all used strains was confirmed. Furthermore, the constructed phylogentic trees (Figures 4 and 5) revealed comparable clustering of the leptospiral strains. In general, intermediate (blue color), pathogenic (red color) and non-pathogenic (green color) strains were clearly separated into different clusters. In addition, pathogenic strains of *L. borgpetersenii* and *L. interrogans* were divided into separate groups*.* Based on the sequence results, *L. kirschneri* was not separated from *L. interrogans* (see Figures 4 and 5). Remarkably, saprophytic strains and intermediate strains allocated to *L. broomii, L. fainei, L. inadai* (genes *icdA, secY, adk, LipL32, LipL41*) and *L. alexanderi* and *L. weilii* (genes *LipL32 and LipL41*) did not produce PCR products for the MSLT data analysis of the genes indicated. Clustering of the MSP Dendrogram (Figure 1) corresponded with the constructed phylogenetic trees (Figures 4 and 5) and confirmed the comparability of mass spectrometry and molecular typing methods.

## Discussion

Recently, it was shown that the optimization and rigorous control of sample preparation are the most critical parameters for successful typing of bacterial strains, using MALDI-TOF MS [34]. To establish a robust extraction procedure for *Leptospira* spp., we optimized the commonly used ethanol/formic acid extraction protocol from Bruker Daltonik GmbH by introducing minor modifications. In this context, Djelouadji et al. demonstrated [27] that reliable leptospiral species identification is possible with directly spotted samples when organisms are available in sufficient numbers (e.g. > 1 x 10^5^ per ml). In our hands, leptospiral cultures needed to reach a minimal concentration of 1 x 10^6^ organisms per ml for a successful extraction procedure. Below this concentration, no visible pellet was found after centrifugation and, following that, results of the extraction procedure were inadequate. As described by Freiwald and Sauer [35], higher densities of bacterial organisms are needed for successful extraction procedure. This might be critical in applying the described procedure in routine diagnostics, since the isolation of *Leptospira* spp. from clinical samples, such as urine or blood, is difficult and time-consuming. It should be emphasized that positive results in laboratory cultivation may take up to six months [3]. However, it was reported that microorganisms in urine (*Escherichia coli*) [36] and in blood samples [37] were identified directly with MALDI-TOF MS.

The inclusion of the optional PBS washing step into the extraction procedure resulted in the lack of protein peaks in the mass range beyond 11,000 Da. Even if this finding did not influence the correct species identification, it can be critical when protein peaks with masses above 11,000 Da are essential for the separation of leptospiral strains. As mentioned above, this emphasizes the need for a standardized preparation procedure to exclude any influence of the sample preparation procedure on the quality of the protein spectra. Other studies also showed that bacterial protein profiles may be altered by varying growing conditions and extraction solvents. For example, triflouroacetic acid can be used instead of formic acid or different matrix solutions can be applied [23,38,39]. To overcome this problem, all leptospiral samples included in this study were cultured and extracted under standardized conditions. Furthermore, as proposed by Welker et al. [40] to ensure the quality of an established protein reference spectra database, each genomospecies was represented by several strains. Beyond this, MSP creation was performed twice, in two self-contained laboratories. The quality of the established database was confirmed by defined measurements. To exclude any influence of the preparation method sample protein extracts of the reference strains were spotted and measured four times in each laboratory. Reliable species identification for all used strains was successful. Only one field isolate, *L. kirschneri* serovar Grippotyphosa, did match with the same score value for *L. kirschneri* and *L. interrogans*. This indicates that the differentiation of closely related species by MALDI Biotyper™ is difficult. In this case, 16S rRNA sequencing revealed the correct species to be *L. kirschneri*. The close phylogenetic relationship of the two species was confirmed in former sequencing projects [41-43]. Nevertheless, a clear separation of the species *L. borgpetersenii* and *L. interrogans* was possible. Studies showed that the genome of the two species *L. interrogans* and *L. borgpetersenii* differ in their chromosome size and gene numbers. In comparison to the other two pathogenic species, *L. borgpetersenii* contains the smallest genome size with 3,931 kb. This pathogenic species is not adapted for the existence in the outer environment [1,44], which may be due to the loss of genes in the evolutionary process. Differences in the bacterial genome structure followed by the transcription of different proteins in the host and under laboratory conditions can result in the loss of protein peaks in MALDI-TOF MS spectra leading to differences in the proteome profiles. This observation is well-described for other microorganisms such as *Brucella* spp. [37,45]. Considering these known leptospiral genomic variations, we hypothesize that it is possible to distinguish lepotspiral strains on the basis of discriminating peaks in their protein profiles. The most critical point for successful subtyping of gram-positive and gram-negative bacteria is the rigorous control of the extraction procedure, as described for *Salmonella enterica*[46]. For this purpose all lepotspiral strains were processed under defined working conditions. The statistical analysis software package ClinProTools was applied in this study. Reproducibility of the data was assured by applying two independently generated datasets of the same strains to ClinProTools analysis. The software automatically processes, recalibrates and compares the loaded spectra using an internal algorithm [47]. The processed peaks are then sorted according to their statistical separation strength [48]. Using this method, we were able to detect differentiating peaks for the serovars used in this study namely *L. interrogans* serovar Pomona and Copenhageni, *L. kirschneri* serovar Grippotyphosa and *L. borgpetersenii* serovar Saxkoebing and Tarassovi (Table 4 and Table 5). Minor discrepancies in the protein profiles were present for the serovars Australis and Icterohaemorragiae. Based on the statistical method PCA, one additional leptospiral strain, *L. borgpetersenii* serovar Sejroe, formed a distant cluster with regard to the other strains (Figure 3). A *L. borgpetersenii* serovar Sejroe specific peak at 6,003 Da was also detected by applying ClinProTools analysis in one of the two datasets. Since it could not be verified by the second dataset, it has not been further considered for identification. No differentiation was observed for the genomospecies *L. kirschneri*. Our findings lead to the conclusion that it is possible to discriminate our applied leptospiral strains on the basis of differences in their protein peak patterns, but we cannot claim this for other serovars or strains. Strain-specific differentiation using MALDI-TOF MS analysis has previously been shown by different studies [49-51] and discrimination of different serovars of *Salmonella enterica* has been postulated before [46,52]. This supports the hypothesis that MALDI-TOF MS is an important and useful technology for the identification and subtyping of bacterial isolates. Serovars of leptospiral strains are determined by antigenic variations in the LPS [15]. MALDI-TOF MS, however, mainly detects ribosomal proteins [45]. Consequently, we cannot claim conclusively that we identified universal serovar-specific peaks since we used a selected panel of serovars in this study. We suppose that the observed peak differences for some strains indicate serovar affiliation. To confirm this finding a larger panel of strains and serovars needs to be tested.

The results of gene sequencing confirmed the MALDI-TOF MS-based species identification of all *Leptospiral* strains. The dendrogram of the reference spectra matched the phylogenetic trees constructed, using16S rRNA sequences and MLST data (Figures 4 and 5). Minimal discrepancies that occurred within single clades can be explained on the basis of the used target genes, since MALDI-TOF MS mainly detects ribosomal proteins [45]. That is why MSPs dendrograms are closely comparable to phylogentic trees based on 16S rRNA sequencing [23,26,35]. From the six genes used for the MLST, only the *rrs2* gene encodes for the 16S rRNA. The lack of amplicons for some target genes is most likely due to the absence of certain genes in some leptospiral strains. Non-pathogenic *leptospiral* strains do not carry genes that encode the outer membrane lipopoproteins *LipL32* and *LipL41*[53]. Similarly, it has been reported that PCR fragments were not producible for intermediate and non-pathogenic strains when they were tested for the s*ecY, adk* and *icdA* genes [43,54]. An additional problem is the quality of the PCR method, since many of them do not amplify genes, even though they are present in the organism. The PCR settings must be optimized for intermediate and non-pathogenic strains [55] and, in a recent study, primers were optimized for all genes to provide greater power for discrimination of *Leptospira* strains [54].

Our method showed that MALDI-TOF MS can be a useful tool to identify cultured leptospiral strains at the species level. This would be of interest to diagnostic laboratories, because internal controls for leptospiral cultures such as for MAT panels are indispensable. Species confirmation by MALDI-TOF MS is faster and more easily applied as compared with other, more elaborate, molecular typing methods which may be complemented by MALDI-TOF MS techniques.

## Conclusions

The protein spectra database established in this study was built on a wide variety of well-defined leptospiral strains that represent the major causative agents of leptospirosis in humans and animals, as well as intermediate and non-pathogenic strains. With our established extraction protocol, we were able to reproducibly detect *Leptospira* species from defined samples as well as from field isolates. Analysis with the software ClinProTools suggested discriminating peaks within the pathogenic species *L. borgpetersenii*, *L. interrogans* and *L. kirschneri*, indicating that it is possible to discriminate certain serovars that belong to the same genomospecies using MALDI-TOF MS. Results of the mass spectrometry analysis and the molecular sequence methods correlated well with each other and confirmed the reliability of MALDI-TOF MS in detecting *Leptospira* species.

## Competing interests

The authors declare that they have no competing interests.

## Authors’ contributions

AR executed the MALDI-TOF MS and MLST experiments, analyzed the data and drafted the manuscript; AS initiated the LGL study, participated in the study design at the LGL and critically revised the manuscript; HR provided the field-collected strains and critically revised the manuscript; IH participated in the design of the study, coordinated the experiments at the LGL, participated in the analysis of the data and drafted the manuscript; IK participated in the design of the study, coordinated the experiments at the LMU, participated in the data analysis and critically revised the manuscript; KG processed the MALDI-TOF MS and ClinProTools experiments, performed the 16S rRNA sequencing and analyzed the data; RK participated in the design of the study as well as in the analysis of the data; RKS coordinated and supervised the study at the LMU Microbiology Department and critically revised the manuscript; UB participated in the study design at the LGL and critically revised the manuscript; VD participated in the MALDI-TOF MS study design at the LMU and critically revised the manuscript; VF initiated the LGL study part, participated in the design of the study at LGL and critically revised the manuscript. All authors read and approved the final manuscript.
